# High-Throughput Acoustic FFPE Proteomics Reveals ATP5IF1-Associated Mitochondrial Alterations in Acral Melanoma

**DOI:** 10.1016/j.mcpro.2026.101620

**Published:** 2026-07-13

**Authors:** Diana Lashidua Fernández-Coto, Marisol Ayala, Ramiro Alonso, Alicia Palmqvist, Ma. Fernanda García, Scarlett F. Balderas-López, Henriett Oskolas, Lukas Christersson, Livia Fulöp, Balazs Szigeti, Victor H. Olivera-Rodríguez, Matilda Marko-Varga, Peter Horvatovich, Johan Malm, Leticia Szadai, Roberto Herrera-Goepfert, Attila Marcell Szasz, Sergio Encarnación-Guevara, György Marko-Varga, Jeovanis Gil

**Affiliations:** 1 Center for Genomic Sciences, National Autonomous University of Mexico, Cuernavaca, Mexico; 2 Department of Translational Medicine, Lund University, Lund, Sweden; 3 Surgical Pathology Department, Instituto Nacional de Cancerología, Mexico City, Mexico; 4 Department of Pathology, National Koranyi Institute of Pulmonology, Budapest, Hungary; 5 Faculty of Science and Engineering, Department of Analytical Biochemistry, University of Groningen, Groningen, the Netherlands; 6 Department of Dermatology and Allergology, Albert Szent-Györgyi Medical School, University of Szeged, Szeged, Hungary; 7 Department of Internal Medicine and Oncology, Semmelweis University, Budapest, Hungary; 8 Department of Biotechnology, College of Life Science and Biotechnology, Yonsei University, Seoul, South Korea; 9 1st Department of Surgery, Tokyo Medical University, Tokyo, Japan

**Keywords:** acral melanoma, adaptive focused acoustics, AI-digital pathology, ATP synthase inhibitor IF1, oxidative phosphorylation, plate-scale FFPE proteomics

## Abstract

Formalin-fixed, paraffin-embedded (FFPE) archives underpin dermatopathology and translational oncology, enabling clinically annotated melanoma cohorts, but cohort-scale proteomics remains limited by labor and variability in upstream processing. We established a plate-scale, acoustic FFPE proteomics workflow and integrated it with AI-assisted digital pathology to support composition-aware molecular profiling from routine sections. The optimized pipeline reduces handling steps, is designed to improve reproducibility, and supports rapid parallel processing in a 96-well format. Deep data-independent acquisition MS of 40 primary melanomas spanning acral lentiginous, lentigo maligna, superficial spreading, and nodular subtypes quantified more than 8200 protein groups and a mean of 5200 proteins per tumor. Proteome profiles resolved melanoma subtypes and defined an acral lentiginous melanoma program enriched for translation/biogenesis and extracellular matrix/adhesion processes with relative depletion of lipid and fatty-acid metabolism and peroxisomal pathways. Supervised feature selection highlighted tenascin, periostin, eukaryotic initiation factor 4A-I, and ADP-ribosylation factor 4 and uncovered selective depletion of ATPase inhibitor, mitochondrial (ATP5IF1), a regulator of mitochondrial ATP synthase, in acral lentiginous melanoma. ATP5IF1 depletion persisted after adjustment for mitochondrial proxies and QuPath-derived tumor content, and coincided with higher glycolysis relative to Complex V. This pathology-integrated, high-throughput FFPE proteomics framework enables scalable retrospective discovery and suggests subtype-specific metabolic alterations consistent with mitochondrial remodeling in an underrepresented melanoma subtype.

## Highlights

• Plate-scale acoustic FFPE proteomics enables rapid 96-well melanoma profiling.
• Workflow optimization yields deep DIA coverage from routine FFPE melanoma sections.
• QuPath-guided pathology supports composition-aware proteomic interpretation.
• Acral melanoma shows translation/ECM enrichment and lipid-metabolism depletion.
• ATP5IF1 depletion in ALM persists after pathology & mitochondrial-proxy adjustment.

## Graphical abstract

**Figure:**
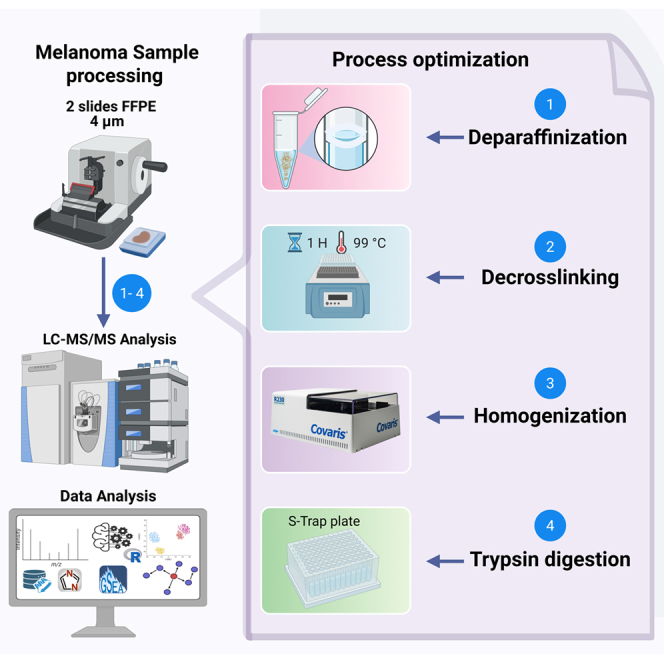

## In Brief

We establish a plate-scale acoustic workflow for FFPE proteomics and integrate it with digital pathology to profile melanoma subtypes. Applied to 40 primary tumors, the workflow resolves acral lentiginous melanoma proteomic features, including translation/ECM enrichment, lipid-metabolism depletion, and ATP5IF1 depletion that persists after adjustment for tumor content and mitochondrial proxies. The study supports scalable, composition-aware discovery from archived melanoma tissue.

Formalin-fixed, paraffin-embedded (FFPE) tissue is foundational to modern histopathology and, by combining preserved tissue morphology with long-term room-temperature storage, constitutes a uniquely valuable resource for retrospective proteomic analyses linked to clinical outcomes and covariates (1, 2). The scale and clinical annotation of contemporary FFPE archives now position (MS-based proteomics to complement histopathology, immunohistochemistry, and genomics by reporting pathway activity and tumor-microenvironment interactions directly in patient cohorts (3, 4, 5, 6).

Realizing this potential at cohort scale remains limited less by MS performance than by the standardization of upstream processing. Conversion of thin FFPE sections into MS-ready peptides requires efficient paraffin removal, reversal of formaldehyde-induced cross-lin ks to restore protease accessibility, and reproducible tissue disruption and solubilization. Mechanistic and experimental studies of fixation chemistry and retrieval kinetics underscore the importance of controlled, high-temperature retrieval and careful handling to recover representative peptide populations from FFPE material (3, 7, 8, 9). Traditional multi-step solvent deparaffinization can further increase hands-on time and variability and may elevate the risk of sample loss when scaled to dozens-to-hundreds of specimens.

Over the past decade, FFPE proteomics has advanced through improved retrieval chemistries, simplified digestion/cleanup formats, including suspension trapping (S-trap), and acquisition/analysis strategies that enable broad proteome coverage from low-input material (1, 2, 10, 11). In parallel, plate-based processing and focused-acoustic homogenization, such as adaptive focused acoustics (AFA) have emerged as practical enablers of high-throughput, reproducible disruption, often a principal bottleneck in large studies, and can be integrated with reduced-solvent or xylene-free deparaffinization approaches compatible with partial automation in clinical research settings (3, 12, 13, 14).

Melanoma provides a stringent and clinically relevant context to evaluate scalable FFPE proteomics because it comprises biologically distinct diseases across anatomic and etiologic settings. In addition to cutaneous melanoma, mucosal and uveal melanomas represent separate entities with distinct molecular drivers and clinical behavior. Within cutaneous melanoma, major histopathologic subtypes include superficial spreading melanoma (SSM), nodular melanoma (NM), lentigo maligna melanoma (LMM), and acral lentiginous melanoma (ALM), which differ in anatomic site, epidemiology, and tumor-stroma context. This heterogeneity motivates subtype-aware molecular profiling and cautions against treating melanoma cohorts as a single class (15). ALM, arising on glabrous skin of palms, soles, and nail units, is relatively more common in non-European populations yet remains underrepresented in many large melanoma datasets derived predominantly from UV-driven cutaneous melanoma, and it is frequently associated with diagnostic delay and outcome disparities (16, 17, 18).

Here, we present and validate a plate-scale FFPE proteomics workflow optimized for cohort studies, integrating one-step deparaffinization, brief high-temperature cross-link reversal, 96-well AFA homogenization, ethanol precipitation, and S-Trap digestion to generate data-independent acquisition (DIA)-ready peptides from low-input tissue sections with reduced hands-on time. Following systematic optimization of deparaffinization chemistry, lysis conditions, and acoustic runtime on test FFPE tissue, we applied the workflow to 40 primary melanomas spanning acral lentiginous, lentigo maligna, superficial spreading, and nodular subtypes. The approach delivered reproducible peptide recovery and deep proteome coverage suitable for subtype-resolved analyses, and it delineated a proteomic phenotype that distinguishes acral melanoma from other cutaneous subtypes. Collectively, these results establish standardized, plate-based FFPE proteomics as a scalable strategy for resolving melanoma heterogeneity, including in underrepresented patient populations, using routinely archived clinical material.

## Experimental procedure

### FFPE Sample Acquisition and Cohort

FFPE tissue samples used for method development and validation were obtained with appropriate institutional approvals. For workflow optimization, archived human trachea FFPE tissue was used as a test model because its cartilage-rich composition provides a stringent extraction challenge.

The clinical melanoma cohort comprised 40 FFPE primary melanoma cases collected at the National Cancer Institute (INCAN), Mexico (approval No. (023/041/PTI) (CEI/012/23)). Four major histological subtypes were represented, with 10 cases each of acral lentiginous, lentigo maligna, superficial spreading, and NMs. For quantitative proteomics, two consecutive 4 μm sections were cut directly from each block into low-binding 1.5 ml tubes. To link proteomic measurements to histopathology and tissue composition, adjacent sections were prepared for hematoxylin and eosin (H&E) staining and reviewed independently by two board-certified pathologists to confirm diagnosis, subtype, and tumor content. Clinical annotations including patient demographics, tumor site, Breslow thickness, ulceration status, stage, and outcome/progression variables where available, were compiled for all cases (Supplemental Table S1: ClinicalData).

### Experimental Design and Statistical Rationale

Workflow optimization experiments were performed using FFPE trachea tissue samples analyzed under multiple experimental conditions with three technical replicates per condition (n = 3) to assess methodological reproducibility. For the clinical cohort analysis, 40 independent FFPE primary melanoma samples representing four histological subtypes were analyzed (n = 10 biological replicates per subtype: acral lentiginous, lentigo maligna, superficial spreading, and NM). Each biological sample was processed once using two consecutive tissue sections combined prior to protein extraction, without additional LC-MS/MS technical replicates. Cohort size and balanced subtype representation were selected to enable exploratory comparative proteomic analyses across melanoma subtypes while minimizing technical variability through standardized sample preparation and DIA-MS acquisition.

### Histopathological Characterization and Digital Pathology

H&E-stained sections from the 40 melanoma cases were evaluated by two certified pathologists to confirm subtype and assess tumor content. Histopathological variables including Breslow thickness, ulceration, mitotic activity, regression, necrosis, and inflammatory infiltrates were recorded. High-resolution whole-slide images were acquired using an automated slide scanner and analyzed in QuPath (19). Digital pathology annotations were used to quantify tissue components (tumor, stroma, inflammatory infiltrates/TIL-enriched regions, regression, and necrosis where present). Quantitative estimates of tumor content, extracellular matrix (ECM), and TIL-enriched regions obtained from QuPath were incorporated into downstream statistical analyses (Supplemental Table S1: QuPath-derived composition). Group-wise comparisons were performed using Kruskal–Wallis tests, followed by multivariate permutational analysis of variance (PERMANOVA) based on Euclidean distance matrices calculated from scaled variables.

### Workflow Optimization Using FFPE Test Tissue

Pre-analytical workflow optimization was performed using FFPE trachea sections (two 4 μm sections per replicate), with each condition evaluated in triplicate. The objective was to systematically evaluate the impact of deparaffinization strategy, lysis buffer composition, and AFA parameters on protein recovery and downstream proteomic performance. Unless otherwise specified, all samples were processed using a common downstream workflow consisting of reduction, alkylation, ethanol precipitation, S-Trap digestion, and LC-MS/MS analysis (see details below).

### Reference Workflows

Two Reference Protocols Were Used as Benchmarks:

(i) *Standard in-house protocol.* Sections were deparaffinized using four cycles of EnVision Target Retrieval Solution (1 × ; Dako; Japan), each consisting of incubation at 99 °C for 10 min with gentle agitation, followed by paraffin removal by centrifugation and aspiration. Samples were then resuspended in TEAB/SDS/DTT buffer (100 mM triethylammonium bicarbonate (TEAB), 5% SDS, and 25 mM DTT) and subjected to heat-induced crosslink reversal (99 °C for 1 h). Tissue disruption was performed using a Bioruptor Plus sonicator (40 cycles; 15 s on/15 s off; 4 °C) prior to downstream processing (1, 20).
(ii) *Manufacturer-recommended Covaris workflow.* FFPE sections were processed without prior deparaffinization using Covaris AFA lysis buffer followed by AFA treatment using the R230 “deparaffinization/homogenization” method in a 96-well format (5 min per column; acoustic parameters as specified by the manufacturer). Samples were then subjected to heat-induced crosslink reversal (99 °C, 1 h) followed by a second AFA homogenization step using identical settings.

### Hybrid and Test Conditions

To decouple the contributions of deparaffinization chemistry, lysis buffer composition, and acoustic processing, the following experimental conditions were evaluated:

(1) **One-step EnVision + AFA workflow.** Sections were subjected to a single EnVision deparaffinization step (99 °C, 10 min), followed by AFA processing as described above. Two buffer conditions were tested: (a) Covaris AFA lysis buffer or (b) TEAB/SDS/DTT buffer.
(2) **Covaris workflow with buffer substitution.** The manufacturer-recommended Covaris workflow was performed as described, except that TEAB/SDS/DTT buffer was used in place of the proprietary AFA lysis buffer.
(3) **AFA runtime titration.** Within the one-step EnVision condition, the duration of the R230 AFA treatment was varied (5, 10, 20, and 40 min per column) while maintaining constant acoustic settings.
(4) **Format comparison (plate *versus* glass vials).** Selected conditions were replicated using Covaris-recommended glass vials, applying the same AFA parameters and a 5-min treatment, to assess potential differences between plate-based and vial-based processing.

Workflow performance was assessed using total protein recovery, peptide yield, peptide/protein identifications by LC-MS/MS, and technical reproducibility across replicates.

### Optimized FFPE Protein Extraction and Digestion Workflow

**Fig. 3:**
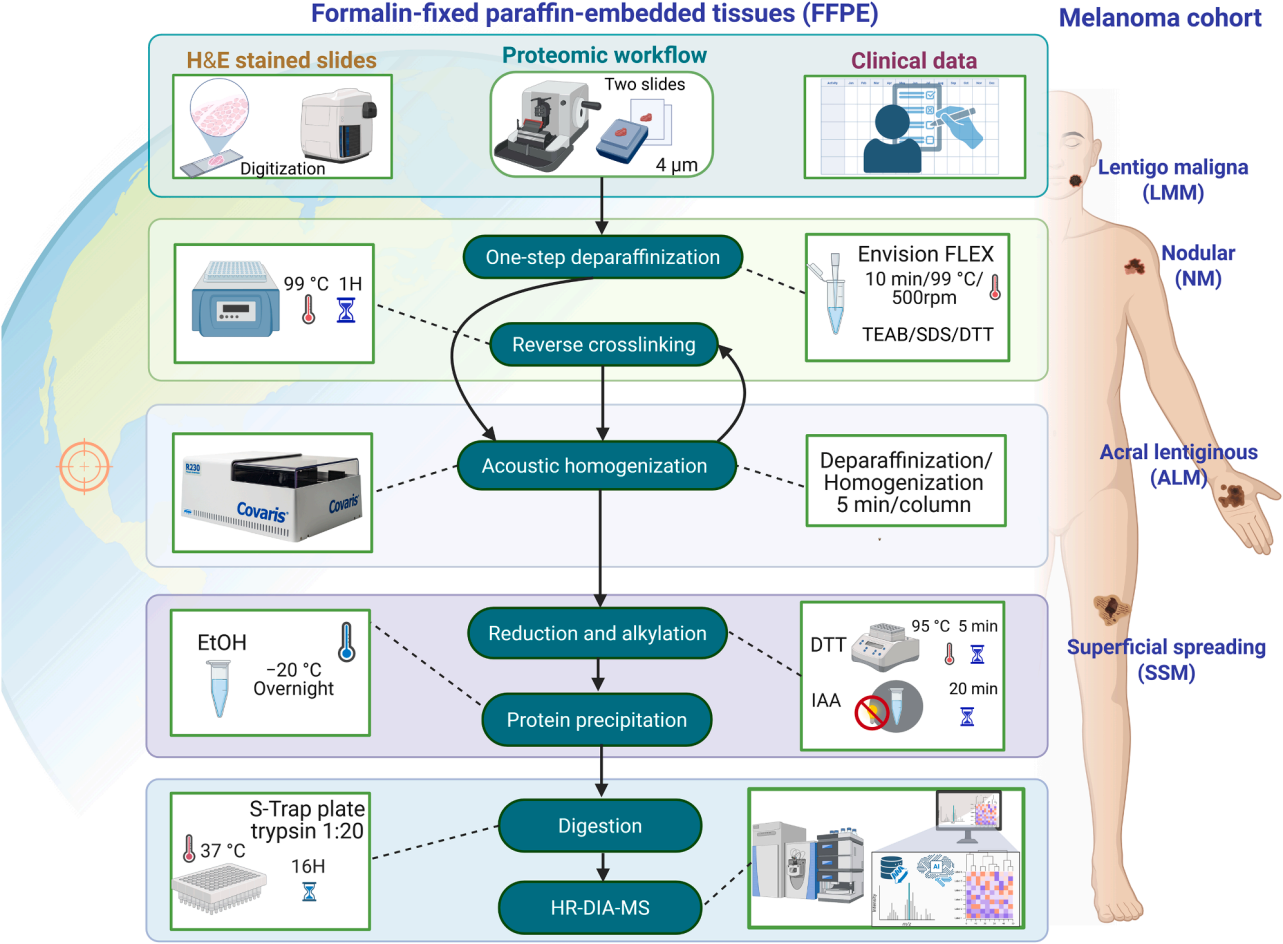
**Optimized FFPE proteomics workflow for plate-scale protein extraction and DIA profiling.** Schematic overview of the Covaris-based FFPE proteomics pipeline integrating one-step EnVision buffer-based deparaffinization, heat-induced cross-link reversal, and acoustic homogenization on the Covaris R230. Proteins are reduced, alkylated, ethanol-precipitated, and processed using suspension trapping 96-well plates for on-column digestion and cleanup. For melanoma cohort profiling, 20 μg proteins per sample was digested, with approx. 80% average peptide recovery after processing, and peptides were analyzed by DIA LC-MS/MS to enable deep, quantitative proteome coverage across 40 tumors.

Based on optimization results, an optimized workflow was established (Fig. 3). Unless otherwise stated, all cohort samples were processed using this protocol.

#### One-Step Deparaffinization, Antigen Retrieval, and Homogenization

FFPE sections (two 4 μm sections collected directly into a 1.5 ml tube) were covered with 1 ml of EnVision FLEX Target Retrieval Solution (1 × ; Dako; Japan) and heated at 99 °C for 10 min with gentle agitation. Samples were centrifuged (14,000 RCF, 3 min, 4 °C) to pellet tissue, and the supernatant was aspirated without disturbing the pellet. The residual paraffin layer was removed after brief re-centrifugation as needed, and remaining supernatant was discarded.

The tissue pellet was resuspended in 100 μl TEAB/SDS/DTT lysis buffer. Samples were transferred to a 96-well AFA-TUBE TPX plate and processed on a Covaris R230 focused ultrasonicator (Covaris, Woburn, MA) using the deparaffinization method. AFA settings were as follows: Duration 5 min per column, Peak Incident Power 350 W, Duty Factor 30%, Cycles per Burst 200 for an Average Incident Power of 105 W, with a 20 °C temperature setpoint. Samples were then heated at 99 °C for 1 h to promote formaldehyde crosslink reversal and lysis (21). Immediately after heating, samples were homogenized by AFA using the same settings described above. Lysates were clarified by centrifugation (1500*g*, 5 min), and supernatants were transferred to fresh tubes.

#### Reduction, Alkylation, and Precipitation

An aliquot corresponding to 20 μg proteins (estimated by detergent-compatible BCA) was heated at 95 °C for 5 min to complete denaturation/reduction. Cysteines were alkylated by addition of iodoacetamide to a final concentration of 50 mM followed by incubation for 20 min at room temperature in the dark. Proteins were precipitated by adding 9 volumes of ice-cold absolute ethanol and incubating at −20 °C for 16 h. Precipitated proteins were pelleted by centrifugation (14,000 RCF, 10 min, 4 °C), washed once with 90% ethanol, briefly air-dried, and resuspended in 50 μl TEAB/SDS solution (50 mM TEAB, 5% SDS).

#### S-Trap Binding and On-Plate Tryptic Digestion

Proteins were processed using an S-Trap 96-well plate (PROTIFI) according to the manufacturer’s workflow with minor adaptations. Briefly, lysates were acidified by adding phosphoric acid to a final concentration of 1.2% (v/v) and then diluted with S-Trap binding buffer (90% methanol in 100 mM TEAB) at a 6:1 (binding buffer:sample) ratio. The resulting mixture was loaded onto the S-Trap plate and centrifuged to bind proteins in the matrix. Wells were washed four times with binding buffer to remove detergent and other contaminants.

On-matrix digestion was performed by adding sequencing-grade modified trypsin in 50 mM TEAB at a 1:20 (trypsin:protein) ratio and incubating overnight at 37 °C. Peptides were eluted sequentially with 0.2% formic acid in water followed by 50% acetonitrile/0.2% formic acid, pooled, and dried in a vacuum concentrator. Dried peptides were stored at −80 °C until LC-MS/MS analysis and reconstituted immediately before injection in 0.1% trifluoroacetic acid (TFA) in 98:2 water:acetonitrile. All samples were processed simultaneously using the same standardized workflow and identical conditions, thereby minimizing potential batch effects and obviating the need for batch correction.

#### LC-MS/MS Analysis

Peptide samples (approximately 1 μg) were analyzed on a Dionex UltiMate 3000 RSLCnano UHPLC system coupled to a Q Exactive HF-X Orbitrap mass spectrometer (Thermo Fisher Scientific, Waltham, MA). Reconstituted peptides were loaded onto a trapping column (Acclaim PepMap100 C18 trap, 3 μm, 100 Å, 75 μm × 2 cm; nanoViper) at 5 μl/min using 0.1% TFA and 2% acetonitrile, and subsequently separated on an analytical nano-column (EASY-Spray PepMap RSLC C18, 2 μm, 100 Å, 75 μm × 50 cm) maintained at 60 °C. Peptides were eluted at 300 nl/min using a 60 min non-linear gradient of mobile phase B (0.1% formic acid in 80% acetonitrile) against mobile phase A (0.1% formic acid in water). The gradient was: 2% to 27% B over 55 min, followed by ramps from 27 to 35% B (2 min) and 35 to 55% B (2 min), then increased to 90% B over 1 min.

The mass spectrometer was operated in DIA mode using variable isolation windows defined empirically based on prior DDA experiments. Each DIA cycle comprised 57 scans, three MS1 survey scans (resolution 120,000 at m/z 200; AGC target 3 × 10ˆ6; maximum injection time 50 ms) distributed every 18 MS2, resulting in an overall cycle time of approximately 1.3 s. In total, 54 variable-width MS2 scans are included in the DIA cycle, spanning m/z 370 to 1460 with 1 m/z overlap between windows (Supplemental Table S2: DIAcycles). MS2 spectra were acquired at 30,000 resolution (at m/z 200) with an AGC target of 1 × 10ˆ6 and automatic injection time, using higher-energy collisional dissociation (HCD) with a normalized collision energy of 28.

### Data Analysis

DIA data were processed using DIA-NN v2.2. Protein identification and quantification were performed at 1% false discovery rate at the precursor level. Searches used an in silico-generated spectral library based on the human UniProt reference proteome including isoforms (accessed July 2025). The generated spectral library contained 42,482 protein isoforms. Carbamidomethylation of cysteine was specified as a fixed modification; methionine oxidation and N-terminal acetylation were specified as variable modifications; trypsin specificity with up to 1 missed cleavage was used. Precursor and fragment mass tolerances were not fixed manually; DIA-NN automatic mass-accuracy optimization was enabled. Optimized precursor mass accuracies typically ranged from 5 to 7 ppm across runs, while fragment ion mass accuracy was handled by DIA-NN’s automatic optimization. Match-between-runs was enabled, and cross-run global normalization was applied prior to downstream analyses. Protein-group intensities were log2-transformed, and proteins were retained for differential analysis only if they contained ≥70% valid values across the dataset (Supplemental Table S2: Processed_Matrix).

Gene set enrichment analysis (GSEA) was performed against Reactome pathways and Gene Ontology Biological Process gene sets, comparing ALM against all other subtypes. Significantly dysregulated functional annotations (FDR <0.05) were visualized in Cytoscape v3.10.3. Machine learning approaches (Random Forest, LASSO regression, and feseR), implemented in R v4.4.2, was applied to identify subtype-discriminative protein signatures. Briefly, proteins were first filtered to retain those with ≥70% valid values across the cohort, and feature selection was performed independently for each method: Random Forest (variable importance based on mean decrease in accuracy, 2000 trees), LASSO (multinomial regression with 5-fold cross-validation, ranking non-zero coefficients at the selected lambda by maximum absolute value), and feseR (workflow-based preprocessing and ranking by maximum absolute one-*versus*-rest correlation), with the top 100 proteins consistently retained in all cases.

**Fig. 5:**
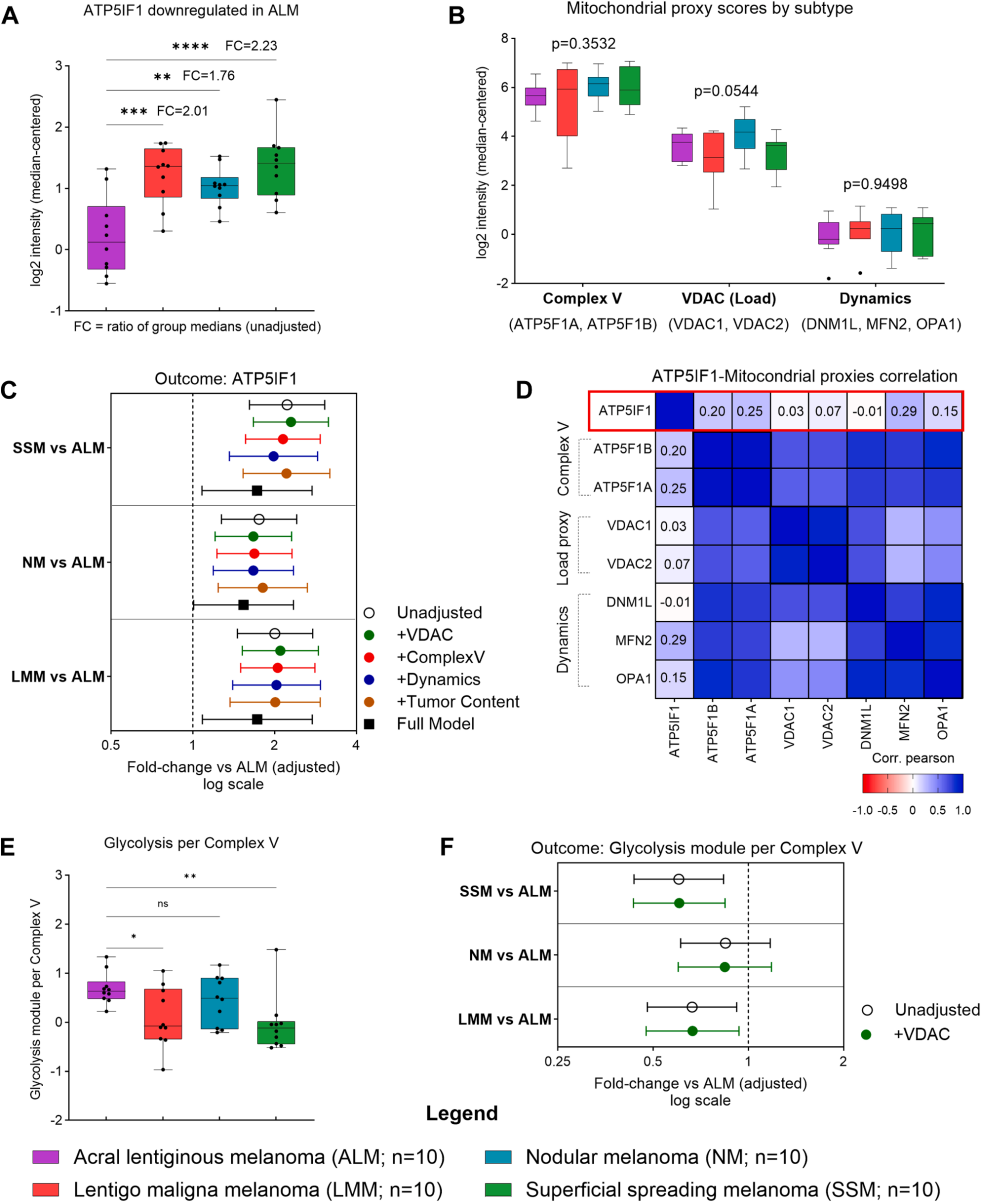
**ATP5IF1 is selectively depleted in acral melanoma independent of mitochondrial load, tumor content, Complex V abundance, and mitochondrial dynamics proxies.***A*, ATP5IF1 abundance across melanoma subtypes. *Boxplots* show ATP5IF1 log2 intensity (median-centered) by subtype; points represent individual tumor samples. Pairwise comparisons versus ALM; significance is denoted by asterisks (∗∗*p*-value<0.01, ∗∗∗<0.001, ∗∗∗∗<0.0001; one-way ANOVA, followed by two-sided multiple-comparison framework corrected with Dunnett test as implemented in Prism). “FC” indicates the unadjusted fold-change computed as the ratio of group medians (subtype/ALM). *B*, composite proxy scores are not subtype-dependent. Composite scores were computed as the mean of median-centered log2 intensities for: Complex V (ATP5F1A, ATP5F1B), VDAC load (VDAC1, VDAC2), and Dynamics (DNM1L, MFN2, OPA1). *p*-values above each composite denote the overall subtype effect (one-way ANOVA test across the four subtypes). *C*, robustness of the ALM ATP5IF1 depletion to covariate adjustment. Forest plot of adjusted fold-changes *versus* ALM (x-axis). Points are fold-changes derived from subtype coefficients, and whiskers represent 95% confidence intervals for each comparison. Models were fitted with ALM as the reference level: Unadjusted (log2(ATP5IF1) ∼ subtype); +VDAC (log2(ATP5IF1) ∼ subtype + VDACscore); +ComplexV (log2(ATP5IF1) ∼ subtype + ComplexVscore); +Dynamics (log2(ATP5IF1) ∼ subtype + Dynamicsscore); +Tumor Content (log2(ATP5IF1) ∼ subtype + %TumorContent); Full model (log2(ATP5IF1) ∼ subtype + VDACscore + ComplexVscore + Dynamicsscore + %TumorContent) where each “score” is the composite defined in (*B*). *D*, ATP5IF1 weakly correlates with proxy proteins. Pearson correlation matrix for ATP5IF1 and the proxy proteins used in (*B*), computed across all tumors using median-centered log2 intensities. The ATP5IF1 row is highlighted (*red outline*), illustrating generally low correlations between ATP5IF1 and mitochondrial protein proxies. *E*, glycolysis module per Complex V across melanoma subtypes. A glycolysis module score was computed as the mean of median-centered log2 intensities for ALDOA, TPI1, ENO1, PGK1, PGAM1, GAPDH, PKM, and LDHA; Glyco_per_ComplexV was calculated as (glycolysis module score − ComplexVscore). *Boxplots* show Glyco_per_ComplexV by subtype with individual tumor samples overlaid. Pairwise comparisons are versus ALM; significance is denoted by asterisks (∗*p*-value <0.05, ∗∗<0.01; one-way ANOVA followed by Dunnett’s test as in *A*). *F*, subtype effects on Glyco_per_ComplexV are independent of mitochondrial load. Forest plot of fold-change *versus* ALM (x-axis; log scale) for Glyco_per_ComplexV showing Unadjusted (Glyco_per_ComplexV ∼ subtype) and +VDAC (Glyco_per_ComplexV ∼ subtype + VDACscore) models. ALM, acral lentiginous melanoma

For targeted ATPase inhibitor, mitochondrial (ATP5IF1) analyses, multiple linear regression models were fitted in Graphpad with ALM as the reference subtype. The primary outcome was median-centered log2 ATP5IF1 intensity. Models included subtype alone or subtype adjusted for VDAC mitochondrial-load score, Complex V score, mitochondrial dynamics score, QuPath-derived tumor content, or all covariates simultaneously. Composite scores were calculated as the mean of median-centered log2 intensities for the proteins indicated in Figure 5. Subtype coefficients were converted to adjusted fold-changes relative to ALM, and 95% confidence intervals were calculated from model standard errors. A similar modeling framework was applied to the glycolysis-module-per-Complex V score, with and without adjustment for VDAC mitochondrial-load score.

Differences in protein expression levels among melanoma subtypes were evaluated using a one-way ANOVA, with subtype as the between-group factor. Protein expression values were analyzed on a log2-transformed, normalized scale. For global subtype comparisons, post hoc pairwise comparisons were conducted using Tukey’s honest significant difference test. For targeted comparisons against ALM, including ATP5IF1 abundance and glycolysis-module-per-Complex V analyses, ANOVA was followed by Dunnett’s multiple-comparisons test using ALM as the reference group. Adjusted *p*-values (p.adj) were used to determine statistical significance. A *p*-value ≤0.05 was considered statistically significant. All analyses were performed in R unless otherwise specified.

### Ethics Statement

FFPE melanoma specimens were obtained from the Instituto Nacional de Cancerología (Mexico City, Mexico) under institutional ethics approval No. (023/041/PTI) (CEI/012/23). The requirement for informed consent was waived by the institutional Ethics and Research Committees because the study used retrospective archival FFPE tissue from the Surgical Pathology archive, posed minimal risk to participants, and all samples/data were handled in an anonymized/de-identified manner prior to proteomic and image-analysis workflows. The study was conducted in accordance with the Declaration of Helsinki and applicable local regulations governing human tissue research.

## Results

### Optimization of a High-Throughput FFPE Extraction Method

Initial comparisons between a conventional FFPE workflow and Covaris-based approaches highlighted key throughput bottlenecks in standard processing. The multi-step protocol, requiring repeated EnVision washes and serial Bioruptor sonication, exceeded 3 h of hands-on/active processing per sample. In contrast, the Covaris R230 96-well plate format enabled parallel processing with substantially reduced hands-on time.

**Fig. 1:**
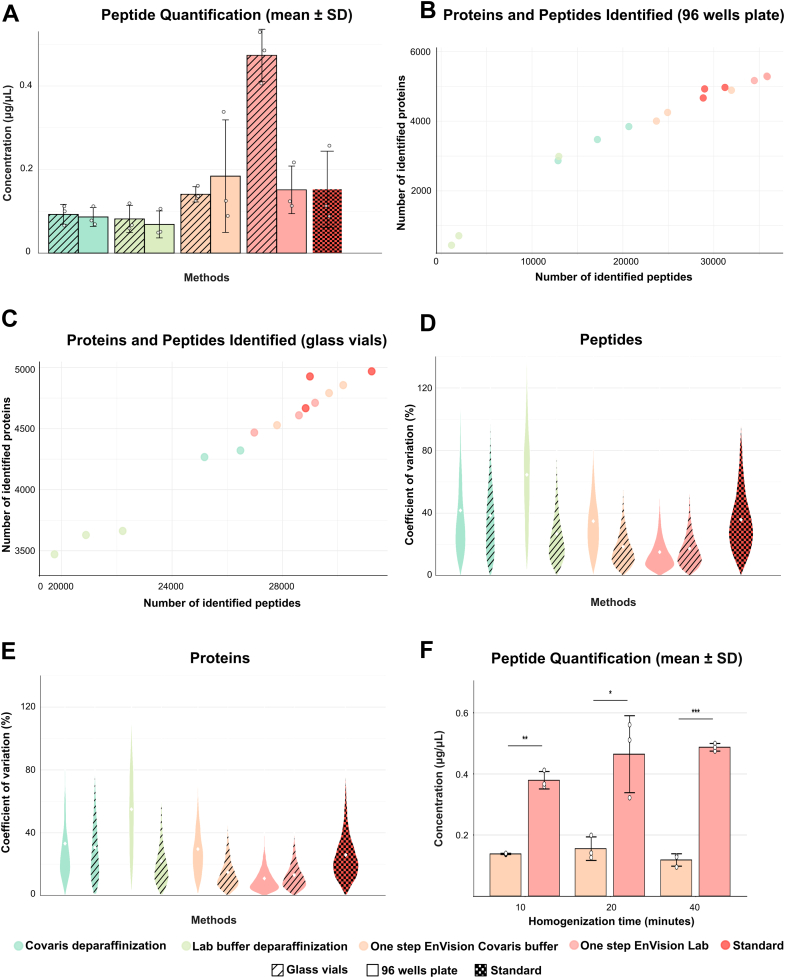
**Optimization of sample processing strategies on Covaris R230.***A*, peptide yield from FFPE trachea processed in the 96-well format and in individual glass vials comparing: (*i*) standard in-house FFPE protocol (multi-step heat-driven deparaffinization using repeated EnVision cycles, followed by high-SDS lysis, heat-induced crosslink reversal, and mechanical disruption by Bioruptor sonication), (*ii*) manufacturer-recommended Covaris workflow (direct AFA-based deparaffinization and homogenization in Covaris lysis buffer using the R230 system, followed by heat-induced crosslink reversal and a second AFA step), (*iii*) covaris workflow with SDS lysis buffer substituted (identical to the manufacturer protocol but replacing the Covaris buffer with a TEAB/SDS/DTT buffer to assess the effect of lysis chemistry), (*iv*) one-step EnVision deparaffinization followed by AFA homogenization in Covaris buffer (hybrid workflow combining simplified single-step deparaffinization with AFA-based disruption and Covaris lysis conditions), and (*v*) one-step EnVision deparaffinization followed by AFA homogenization in SDS lysis buffer (hybrid workflow combining simplified deparaffinization with detergent-based lysis and AFA processing to evaluate combined effects on extraction efficiency). *B* and *C*, peptide and protein group identifications by DIA-MS for samples processed in (*B*) 96-well plates and (*C*) glass vials under the tested conditions. *D and E*, technical variability across replicates expressed as CV at the (*D*) peptide and (*E*) protein-group level (white point denotes the mean). *F*, effect of AFA homogenization time (10, 20, and 40 min per column) on peptide yield for one-step EnVision deparaffinization performed with Covaris versus SDS lysis buffers. Data are shown as mean ± SD. Statistical significance: ∗*p* < 0.05, ∗∗*p* < 0.01, ∗∗∗*p* < 0.001. AFA, adaptive focused acoustics

Using FFPE trachea sections as a test tissue, we systematically optimized deparaffinization, lysis conditions, and acoustic homogenization to maximize yield and reproducibility. 10 minutes of AFA homogenization in the 96-well format yielded extraction efficiency comparable to our standard in-house FFPE protocol (high-SDS lysis with Bioruptor sonication), demonstrating that plate-based high-throughput processing can match conventional performance while enabling parallel sample handling (Fig. 1*A*). For deparaffinization, a single-step EnVision treatment performed comparably to the labor-intensive multi-wash protocol while reducing handling and sample stress. In contrast, omitting chemical deparaffinization (lysis buffer plus sonication only) led to poor homogenization consistent with residual paraffin and reduced recovery with both Covaris and SDS lysis buffers, establishing one-step EnVision as the minimal effective compromise between speed and performance. Although one-step EnVision combined with SDS lysis in glass vials produced the highest yields in some comparisons, the required handling reduced practicality for large cohorts.

Collectively, the optimized workflow -built around one-step EnVision deparaffinization combined with SDS-based lysis under the same plate-scale AFA conditions-outperformed both our standard in-house protocol and the manufacturer-recommended Covaris workflow. Peptide yield and total protein recovery increased by approximately 1.5-fold, consistent with improved lysis and reduced handling losses. The number of unique proteins identified increased by 35% relative to the recommended Covaris workflow and by 8% relative to the standard protocol, while maintaining technical reproducibility (Fig. 1*B*, *D–E*). Similar performance trends were observed in glass-vial processing, where one-step EnVision deparaffinization with either Covaris or SDS buffer, as well as the standard sonication protocol, yielded the highest peptide and protein identifications (Fig. 1*C*).

The optimized workflow also reduced processing time at batch scale. From deparaffinization through ready-to-digest protein pellets, we achieved approximately 3 h of total processing time for 96 samples, whereas the conventional approach would require >30 h if performed serially or substantial manual effort to parallelize. To assess robustness, we quantified technical variability across replicates. One-step EnVision conditions consistently produced lower CVs than alternative approaches, with the one-step EnVision + SDS lysis condition showing low CVs in both glass vials and 96-well plates (Fig. 1, *D–E*).

We then optimized acoustic runtime within the selected chemistry. Comparing one-step EnVision deparaffinization using Covaris versus SDS lysis buffers across homogenization times (10–40 min) showed consistently higher peptide recovery with SDS lysis (Fig. 1*F*), supporting its selection for downstream analyses. Finally, varying AFA sonication time demonstrated diminishing returns beyond short runtimes: extending sonication from 5 to 40 min per column did not materially increase homogenization, protein yield, or peptide/protein identifications. A 5-minute-per-column program recovered a similar number of peptides as 10 min, indicating that most extractable material was recovered within 5 min under these conditions. Accordingly, we selected 5 min per column as the optimal trade-off between yield and throughput, enabling homogenization of a full 96-well plate in approximately 1 h and doubling throughput relative to 10-min runs.

### Histopathological Characterization of Melanoma Samples

Histopathologic review confirmed that all cases represented well-defined melanoma subtypes and were suitable for downstream proteomic profiling. Two board-certified pathologists independently verified diagnosis and subtype assignment and guided selection of tumor-rich regions for molecular processing. The cohort comprised superficial spreading, nodular, lentigo maligna, and acral lentiginous melanomas (n = 10 each).

**Fig. 2:**
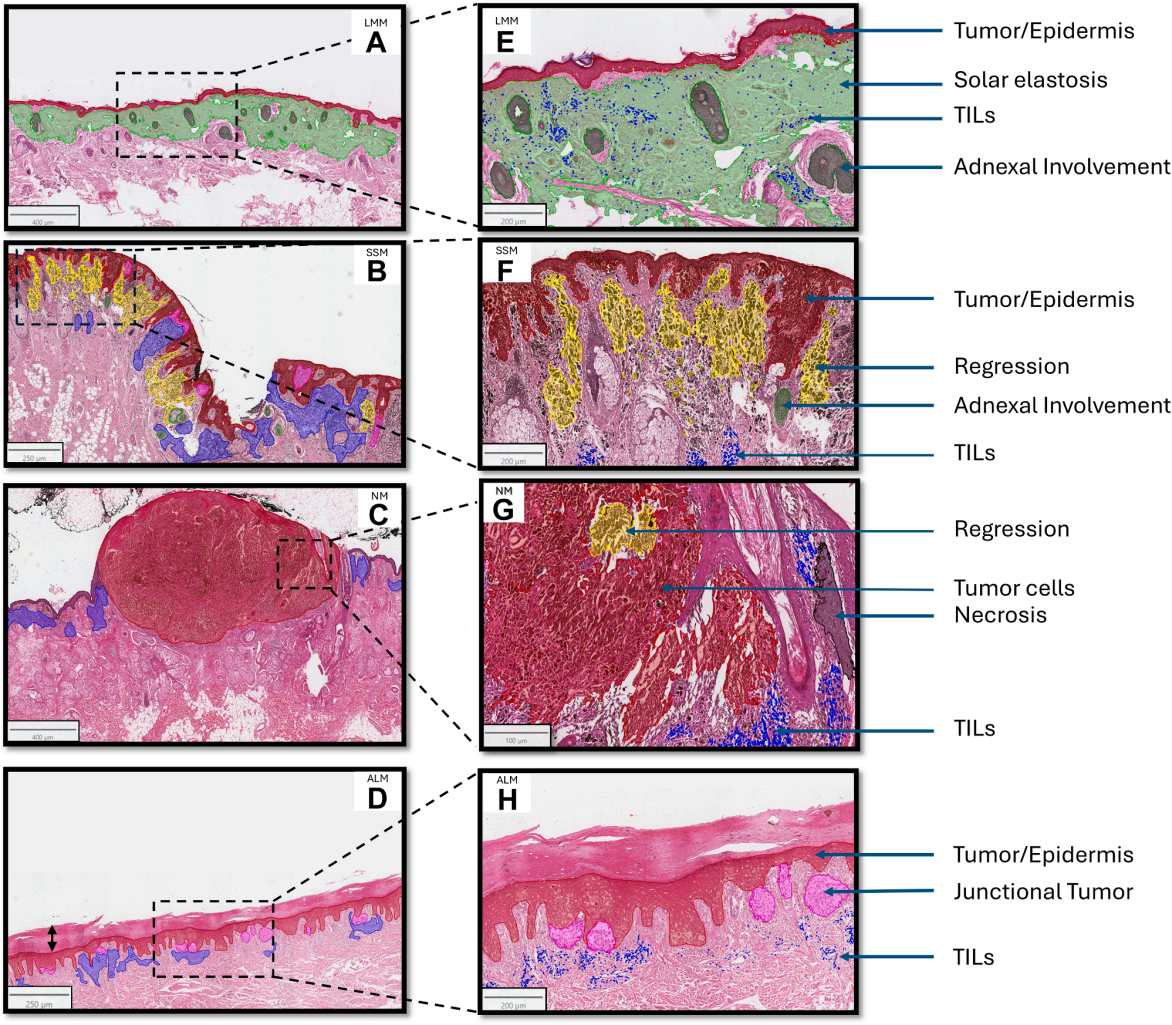
**Representative histopathologic features and QuPath-based computational annotations across melanoma subtypes.** Low- and high-magnification H&E images are shown for lentigo maligna melanoma (*A* and *E*,), superficial spreading melanoma; *B* and *F*, nodular melanoma (*C* and *G*, and (ALM; *D* and *H*,). *Dashed boxes* in low-magnification panels indicate the regions shown at higher magnification. QuPath annotations delineate tumor/epidermal compartments (*red*), junctional tumor regions (*pink*), tumor-associated lymphocytic infiltrates (*blue*), regression (*yellow*), solar elastosis (*light green*), adnexal involvement (*green*), and necrosis (*black*). The *arrow* in *panel D* highlights the relative thickness of the acral stratum corneum. ALM, acral lentiginous melanoma

To standardize tissue interpretation and enable quantitative estimates of histologic composition, whole-slide H&E images were analyzed using QuPath. A consistent annotation scheme was applied across subtypes to delineate the principal tumor compartment and key contextual features relevant to bulk proteomics, including tumor-associated lymphocytic infiltrates, regression, necrosis, adnexal involvement, and solar elastosis. Representative low- and high-magnification fields illustrate both the characteristic morphology of each subtype and the computational annotations used for downstream integration with proteomic measurements (Fig. 2). Dashed boxes in the low-magnification panels indicate the regions shown at higher magnification.

In the representative panels, the main tumor/epidermal compartment is outlined in red, with junctional extension highlighted separately where applicable; tumor-associated lymphocytic infiltrates are marked in blue and regression in yellow. LMM shows prominent solar elastosis (light green) with lentiginous growth and adnexal extension (Fig. 2, *D–E*). SSM demonstrates a broad intraepidermal component with areas of regression and associated inflammatory infiltrates, with adnexal involvement also apparent in the illustrated case (Fig. 2, *B* and *F*). NM is characterized by an expansile dermal tumor nodule with focal necrosis (black) and focal regression/inflammatory features (Fig. 2, *C* and *G*). ALM displays an acral epidermal context with thick stratum corneum (arrow in Fig. 2*D*) and a conspicuous junctional tumor component (Fig. 2, *D* and *H*). Collectively, this histopathologic and computational framework provides an explicit tissue context for interpreting subtype-associated proteomic signatures.

To connect histology with proteomics, proteomic scrolls were cut between two adjacent H&E sections. QuPath-derived compartment annotations from the flanking H&Es (tumor, stroma, immune/TIL-enriched regions, regression, and necrosis) provided composition estimates for the MS-based profiled material. These features varied across cases and subtypes, highlighting the importance of tissue context when interpreting bulk FFPE proteomes.

Across the cohort, Breslow thickness differed significantly among melanoma subtypes (Kruskal-Wallis *p* = 0.0016). ALM exhibited the greatest median thickness (12 mm), followed by NM (5 mm), while SSM and LMM displayed much thinner lesions (0.65 mm and 0.85 mm, respectively). Ulceration was highly prevalent in ALM and NM (80% in each), whereas most SSM and LMM tumors were non-ulcerated (10% and 20%, respectively). Mitotic activity did not differ significantly between subtypes (*p* = 0.93), although ALM tended to exhibit higher rates. Additional features such as regression and brisk tumor-infiltrating lymphocytes were more commonly observed in SSM, while necrosis was identified predominantly in NM and satellite lesions appeared mainly in ALM. Overall, this integrative histopathological assessment provided a robust framework for validating tissue composition, enabling the accurate interpretation of proteomic signatures according to melanoma subtype-specific features.

To determine whether tissue composition differed significantly across melanoma subtypes, quantitative estimates of tumor content, ECM, and TIL-enriched regions derived from QuPath annotations were analyzed statistically. Individual comparisons using Kruskal–Wallis tests did not identify significant subtype-associated differences for tumor content (*p* = 0.162), ECM (*p* = 0.494), or TIL-enriched regions (*p* = 0.402). In addition, multivariate PERMANOVA integrating all three parameters simultaneously did not reveal significant global differences in tissue composition among melanoma subtypes (F = 1.295, R^2^ = 0.108, *p* = 0.239). These findings suggest that the proteomic differences observed across melanoma subtypes are unlikely to be primarily driven by major differences in tissue composition. Quantitative pathology measurements together with the associated statistical analyses are provided in Supplemental Table S1 (Kruskal_Dunn_Results, PERMANOVA).

### Proteomic Profiling of Melanoma FFPE Cohort

Using the optimized 96-well Covaris R230 workflow, we implemented an integrated clinical molecular profiling pipeline for 40 primary melanoma FFPE blocks (10 per subtype), in which proteomic sections were bracketed by H&E slides for AI-digital pathology. This design enabled quantitative proteomics from well-annotated regions while providing composition estimates for the profiled material based on adjacent, computationally annotated sections. Across the cohort, DIA profiling yielded 8219 protein groups in total and an average of 5200 protein groups per tumor (Supplemental Table S2: Protein_matrix), with high quantitative consistency supporting subtype-resolved comparisons (Fig. 3).

**Figure S1:**
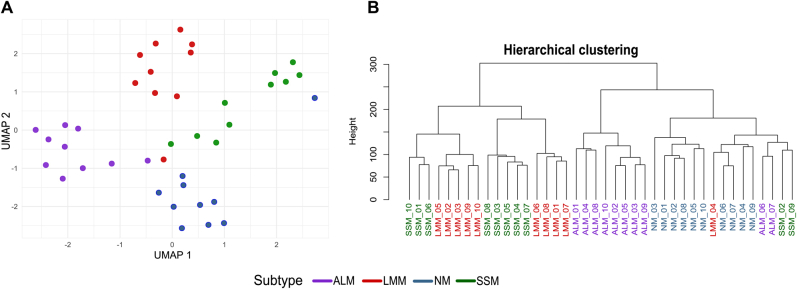
**Unsupervised analysis of proteome -wide melanoma profiles.***A*) UMAP projection of proteomic profiles from melanoma tumors based on proteome-scale quantitative measurements. Data were centered and scaled after missing value imputation, and proteins with zero variance were excluded. UMAP was performed using Euclidean distance with 10 nearest neighbors and a minimum distance of 0.2. Each point represents one tumor sample and is colored according to histopathologic subtype: acral lentiginous melanoma (ALM, *purple*), lentigo maligna melanoma (LMM, *red*), nodular melanoma (NM, *blue*), and superficial spreading melanoma (SSM, *green*). *B*) Unsupervised hierarchical clustering of melanoma samples using the same scaled proteomic data. Pairwise Euclidean distances between samples were calculated and clustering was performed using Ward’s linkage. Sample labels are colored according to histopathologic subtype.

To standardize cohort-level quantification, we digested a fixed protein input per sample and recovered approx. 80% of peptides on average, supporting consistent loading across tumors. We next assessed whether proteome-scale measurements resolved melanoma subtype structure. Unsupervised analyses using UMAP and hierarchical clustering revealed separation of tumors that was broadly consistent with histopathologic subtype, indicating that the proteome captured subtype-associated biological variation (Fig. S1). Given the clinical relevance and relative underrepresentation of acral lentiginous melanoma in many melanoma cohorts, we focused downstream pathway analyses on ALM relative to the remaining subtypes.

**Fig. 4:**
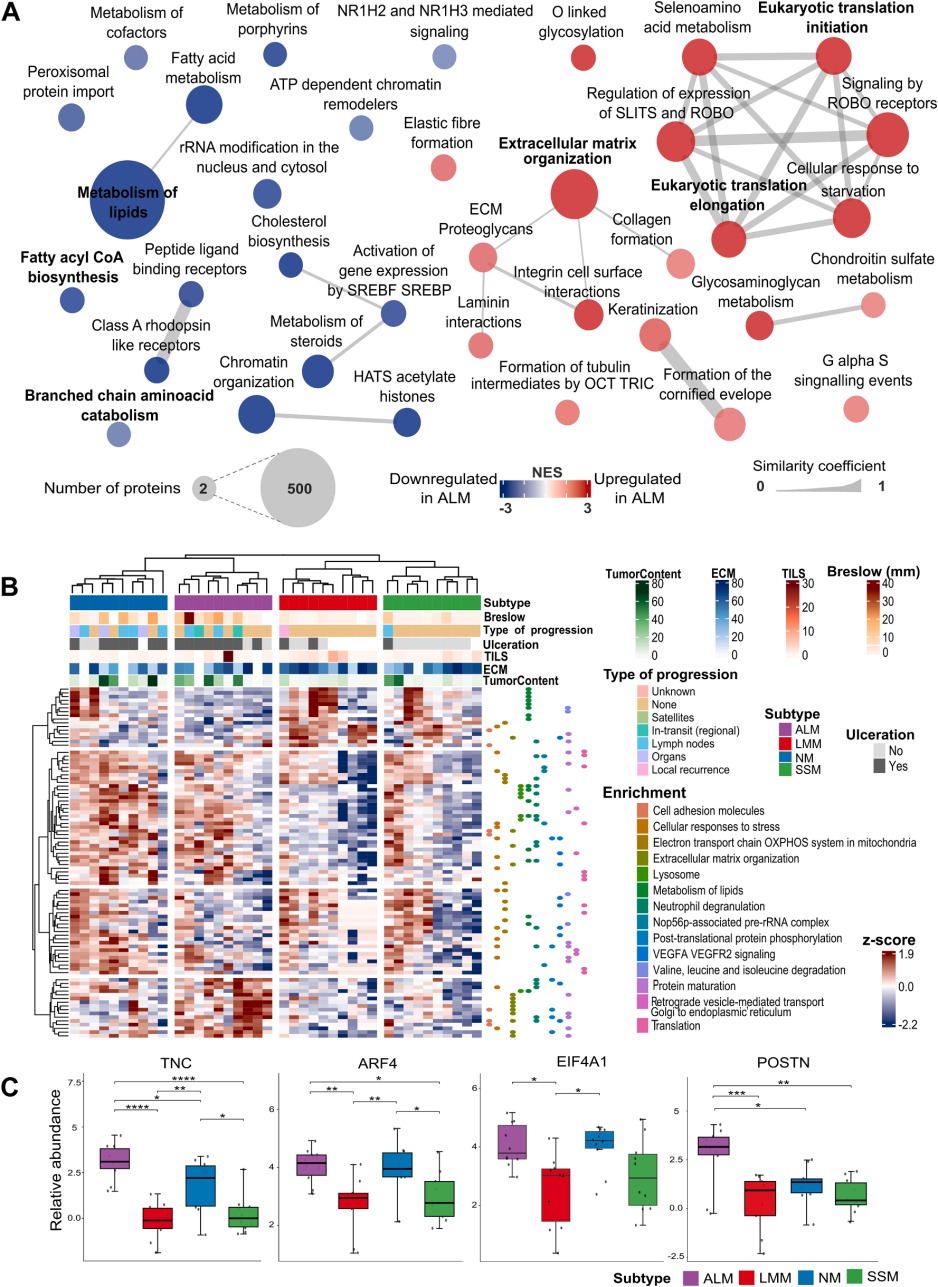
**Proteomic and computational dissection of melanoma subtype-associated signatures.***A*, enrichment map summarizing GSEA results comparing acral lentiginous melanoma to non-acral melanomas. Nodes represent gene sets (size proportional to number of proteins; *color* indicates normalized enrichment score), with edges indicating overlap/similarity between sets. ALM shows coordinated positive enrichment of translation-associated programs and extracellular matrix/adhesion biology, with negative enrichment of lipid and fatty-acid metabolic pathways. *B*, heat map of subtype-discriminative proteins selected by supervised feature selection/classification approaches (Random Forest, LASSO regression, and feseR). Samples are hierarchically clustered and annotated with histological subtypes and clinicopathologic variables. The functional category key indicates representative biological modules captured within the discriminative feature set. *C*, boxplots showing normalized abundance of TNC, ARF4, EIF4A1, and POSTN across subtypes. These proteins are elevated in ALM and nodular melanoma relative to lentigo maligna melanoma and superficial spreading melanoma and are consistently prioritized among the *top* subtype-discriminative features across models. Each *dot* represents an individual sample. Differences among subtypes were first assessed using a one-way (ANOVA. When the global ANOVA was significant, pairwise comparisons were performed using Tukey’s honest significant difference post hoc test with adjustment for multiple comparisons. Significance levels are indicated as follows: ∗∗∗∗*p* ≤ 0.0001, ∗∗∗*p* ≤ 0.001, ∗∗*p* ≤ 0.01, and ∗ *p* ≤ 0.05. Only pairwise comparisons with adjusted *p*-values ≤0.05 are shown.

GSEA comparing ALM to non-acral melanomas identified a coherent, modular subtype-associated signature (Fig. 4*A*). The enrichment map highlighted a densely connected cluster of positively enriched pathways centered on protein synthesis, including eukaryotic translation initiation and elongation, together with related stress-response and signaling terms (cellular response to starvation and SLIT/ROBO signaling). A second positively enriched module comprised extracellular and adhesion biology, including extracellular matrix organization, collagen formation, integrin cell-surface interactions, laminin interactions, and ECM proteoglycans, alongside glycan-related terms. In contrast, ALM showed a coordinated pattern of negative enrichment dominated by metabolic pathways, most prominently metabolism of lipids, fatty-acid metabolism, including fatty acyl-CoA biosynthesis, and broader lipid regulatory processes, together with cholesterol/steroid metabolism and peroxisomal protein import. Additional negatively enriched terms included branched-chain amino acid catabolism and chromatin-related programs like chromatin organization and ATP-dependent chromatin remodeling, indicating that multiple functional axes differed systematically between ALM and the remaining cutaneous subtypes in this cohort.

To refine subtype-discriminative features beyond pathway-level enrichment, we applied supervised feature selection and classification approaches (Random Forest, LASSO regression, and feseR). The resulting protein panels produced a robust subtype-resolved structure (Supplemental Table S2: SelectedGenesML). Hierarchical clustering of the selected features separated tumors into major branches that were largely concordant with histopathological subtype, with ALM, NM, LMM, and SSM forming distinct sample groupings on the heat map (Fig. 4*B*). Overlay of clinicopathologic annotations on the same clustering revealed that samples in the ALM and NM branches were enriched for higher Breslow thickness and ulceration, whereas LMM and SSM clusters were dominated by thinner, predominantly non-ulcerated tumors; progression annotations with nodal and organ involvement also occurred more frequently among ALM/NM cases in this cohort (Fig. 4*B*).

Functional annotation of the discriminative feature set indicated that the selected proteins were not random but instead organized into coherent biological modules represented across the heat map, including categories related to translation, extracellular matrix organization/cell adhesion, vesicle-mediated transport and Golgi-ER trafficking, mitochondrial electron transport (OXPHOS), lipid metabolism, lysosomal pathways, and immune-associated processes (Fig. 4*B*). This feature-level architecture was consistent with, and extended beyond, the pathway trends captured by GSEA.

Among the most consistently prioritized discriminators across models, Tenascin (TNC) and Periostin (POSTN) showed the strongest elevation in ALM and were also increased in NM relative to LMM/SSM, while Eukaryotic initiation factor 4A-I (EIF4A1) and ADP-ribosylation factor 4 (ARF4) were enriched in the ALM/NM groups compared with LMM and, to a lesser extent, SSM (Fig. 4*C*). These proteins were repeatedly selected across Random Forest, LASSO, and feseR, supporting their robustness as potential candidate markers of subtype-associated proteomic states in this cohort.

### Stoichiometric Depletion of ATP5IF1 Distinguishes Acral Melanoma

ATP5IF1, an endogenous regulator of the mitochondrial ATP synthase, was consistently selected across supervised models as a strong discriminator of melanoma subtype, based on protein group-level quantification supported by a non-isoform-specific peptide (Supplemental Table S2: Peptide_matrix). We therefore performed targeted follow-up analyses to determine whether subtype separation reflected ATP5IF1-specific regulation versus global differences in mitochondrial content, Complex V abundance, or mitochondrial dynamics. Across the study cohort, ATP5IF1 abundance was significantly reduced in ALM relative to the other major subtypes (Fig. 5*A*). To test whether this signal could be explained by mitochondrial abundance or related mitochondrial programs, we summarized mitochondrial load, Complex V core, and dynamics/cristae-associated proteins as composite proxy scores. Non-selective voltage-gated ion channel VDAC1 and VDAC2 were used as surrogates of mitochondrial content due to their abundant localization in the outer mitochondrial membrane, ATP synthase F(1) complex subunit alpha, mitochondrial (ATP5F1A) and ATP synthase F(1) complex subunit beta, mitochondrial (ATP5F1B) as representative core ATP synthase (Complex V) subunits, and dynamin-1-like protein (DNM1L), mitofusin-2 (MFN2), and dynamin-like GTPase OPA1, mitochondrial (OPA1) as markers of mitochondrial fission, fusion, and cristae organization, respectively. These proxy scores showed no significant subtype dependence (Fig. 5*B*).

We next fitted linear models with ALM as the reference level to quantify subtype effects on ATP5IF1 after covariate adjustment. Because bulk FFPE proteomes can be influenced by tissue composition, we included QuPath-derived tumor content as an additional pathology covariate together with mitochondrial proxy scores. ATP5IF1 depletion in ALM remained evident in models adjusted individually for mitochondrial load, Complex V abundance, mitochondrial dynamics proxies, or tumor content, and also in the full model including all covariates simultaneously (Fig. 5*C*). Covariate terms were not consistently informative, indicating that the reduced ATP5IF1 signal was not explained by tumor purity, mitochondrial abundance, Complex V levels, or broad mitochondrial dynamics proxies. In line with these findings, ATP5IF1 showed generally weak correlations with proxy proteins across the cohort (Fig. 5*D*), supporting that ATP5IF1 variation is not simply tracking mitochondrial load or broad mitochondrial remodeling. Finally, to assess whether ALM shows coordinated shifts in energy metabolism relative to respiratory machinery, we calculated a glycolysis module score and expressed it relative to Complex V. Glycolysis module per Complex V differed by subtype and remained associated with subtype after adjustment for mitochondrial load, with LMM and SSM significantly lower than ALM (Fig. 5, *E* and *F*). Together, these analyses suggest a subtype-associated pattern in ALM characterized by ATP5IF1 depletion that is independent of tumor content and mitochondrial load, Complex V abundance, and mitochondrial dynamics proxies, and accompanied by higher glycolytic protein representation relative to Complex V.

## Discussion

FFPE proteomics has progressed from feasibility studies to cohort-scale profiling, with workflows now quantifying thousands of proteins from routine clinical material, including slide-compatible approaches operating directly on histopathology sections (4, 6). In this landscape, a practical bottleneck for many translational dermatology cohorts is increasingly upstream: scalable, reproducible pre-analytics (deparaffinization, decrosslinking, and tissue disruption) that can be executed with low operator burden while preserving quantitative fidelity. We present a workflow that addresses this operational gap by streamlining handling, supporting rapid parallel processing in 96-well format, and delivering deep proteome coverage from routine melanoma sections (>8200 protein groups quantified in total; 5200 proteins per tumor on average). In practice, this combination of throughput and depth enables systematic subtype-resolved discovery in archived, clinically heterogeneous cohorts rather than one-off feasibility demonstrations.

A second practical strength is the integration of quantitative proteomics with computational digital pathology. Proteomic sections were bracketed by H&E slides and annotated in QuPath to guide region selection (22, 23, 24), and adjacent computational annotations provided composition-aware context. This is particularly relevant in melanoma, where tumor purity, stromal remodeling, immune infiltration, regression, and necrosis can vary substantially across cases and may confound bulk molecular readouts if tissue composition is not considered explicitly. In this setting, pathology-informed sampling and interpretation directly improve biological interpretability of cohort-scale proteomes.

Beyond enabling, our subtype-resolved proteome reinforces ALM as a biologically distinct melanoma entity rather than simply “cutaneous melanoma in a different location.” Whole-genome analyses have shown that ALM differs from UV-driven cutaneous melanoma by mutational processes and a comparatively stronger contribution of structural variation/copy-number alteration, supporting subtype-resolved molecular profiling (25). Recent single-cell and spatial multi-omic studies further emphasize acral-specific tumor microenvironments and tumor-immune interactions, including invasion/dissemination-associated programs (26). In this context, our proteomic data characterize a coherent ALM-associated proteomic pattern marked by coordinated enrichment of translation/biogenesis processes (including eukaryotic initiation/elongation and related RNA-processing terms) together with an extracellular matrix/adhesion module. Concomitantly, ALM showed coordinated depletion of lipid and fatty-acid metabolic pathways, including fatty acyl-CoA biosynthesis and broader lipid regulatory processes, alongside peroxisomal import and additional metabolic axes. Supervised feature selection provided protein-level anchors consistent with these modules: TNC and POSTN support an ECM- and niche-associated program linked to aggressive states and microenvironmental remodeling in melanoma models (27, 28, 29), while EIF4A1 links directly to translation initiation and is directionally consistent with functional evidence that eIF4A1/eIF4E-dependent translation promotes melanoma proliferation and invasion (30, 31, 32). ARF4 enrichment is likewise consistent with established roles of ARF-family proteins in membrane trafficking and integrin recycling relevant to migration and invasion (33). While these proteins should not be interpreted as melanoma-specific drivers on the basis of this study alone, they provide convergent protein-level support for the dominant ALM-associated modules observed in the pathway analysis and highlight interpretable candidate markers for follow-up.

A key new mitochondrial insight from the subtype-resolved proteome is the selective depletion of ATP5IF1 in ALM (Fig. 5). Importantly, this effect persisted after adjustment for QuPath-derived tumor content, mitochondrial load, Complex V abundance, and proxies of mitochondrial dynamics/cristae remodeling, and ATP5IF1 correlated weakly with these proxy proteins. Together, these observations are not consistent with a simple “less mitochondria” explanation and instead suggest subtype-associated stoichiometric differences of ATP synthase regulation, specifically a reduced ATP5IF1-to-Complex V relationship in ALM. ATP5IF1 encodes IF1, a pH-sensitive inhibitor of F1Fo-ATP synthase that limits ATP hydrolysis when the enzyme runs in reverse and has also been implicated in ATP synthase supramolecular organization and cristae architecture (34, 35). In parallel, ALM showed a higher glycolysis-module-per-Complex V metric that remained subtype-associated after controlling for mitochondrial load. Although abundance-based proteomics cannot resolve flux, enzyme directionality, or membrane potential, the combination of lower IF1 stoichiometry and relatively higher glycolytic protein representation per Complex V is suggestive of a bioenergetic configuration in which ALM may rely more strongly on cytosolic ATP production in the setting of altered ATP synthase regulation. One mechanistic hypothesis is that reduced ATP5IF1 could lower restraint on reverse-mode ATP synthase activity under respiratory limitation, potentially enabling mitochondria to consume glycolytic ATP to sustain mitochondrial membrane potential Δψm; an established “ATP-hydrolysis-supported Δψm maintenance” strategy in contexts of impaired electron transport or oxygen limitation (34, 36). Such a configuration may support continued mitochondrial protein import, metabolite exchange, and biosynthetic signaling during fluctuating perfusion/nutrient availability and invasive growth, conditions that could be relevant in acral tumor ecosystems. At the same time, this model predicts liabilities: increased dependence on glycolytic ATP supply and on ATP synthase/adenine nucleotide transport capacity. These predictions are testable and motivate functional follow-up in ALM models, including oxygen consumption rate profiling, Δψm stability assays, oligomycin sensitivity under respiratory stress, and orthogonal validation of ATP5IF1 regulation at transcript and protein levels.

The translational value of this work is therefore twofold. First, the pathology-integrated, plate-scale workflow provides a practical route to composition-aware, cohort-scale proteomic discovery in archived melanoma tissue, enabling systematic interrogation of underrepresented subtypes such as ALM. Second, the ATP5IF1 signal highlights a mitochondria-centered candidate signal associated with ALM-specific metabolic alterations. Limitations should be acknowledged: the cohort size (n = 10 per subtype) supports discovery but limits biomarker claims and effect-size precision and proxy-based covariate adjustment does not substitute for direct measurements of respiration, Δψm, or Complex V directionality. Next steps include larger multi-center FFPE validation and targeted, compartment-aware assays coupled to functional bioenergetics to determine whether ATP5IF1 depletion represents a reproducible feature of ALM biology.

In summary, we establish a scalable, pathology-integrated FFPE proteomics workflow that resolves subtype-associated proteomic states in melanoma and identifies ALM as a distinct pathway- and feature-level state. Within this state, ATP5IF1 depletion, which is independent of tumor content, mitochondrial load, Complex V abundance, and dynamics proxies, together with increased glycolysis relative to Complex V provides evidence for a new, testable hypothesis for ALM-specific mitochondrial alterations consistent with mitochondrial remodeling.

## Data Availability

Proteomics data generated in this study were deposited to the ProteomeXchange Consortium via the PRIDE partner repository. The accession number is PXD074156.

## Supplemental data

This article contains supplemental data.

## Confilict of Interest

The authors declare no competing interests.
