# Supplementary material for: High-Throughput Acoustic FFPE Proteomics Reveals ATP5IF1-Associated Mitochondrial Alterations in Acral Melanoma
Source: Mol Cell Proteomics. 2026 Jul 13;25(8):101620. doi: 10.1016/j.mcpro.2026.101620 (PMC13452992; doi:10.1016/j.mcpro.2026.101620)
Supplement: Supplementary file 1 [file mmc3.docx]

**Supplementary Table S1. Clinical and histopathological information, digital pathology measurements, and statistical analyses of the melanoma discovery cohort.**

**Sheet 1. ClinicalData**

Clinical and histopathological information for the 40 melanoma cases included in the proteomic discovery cohort. Each row corresponds to one patient/sample, and columns include demographic characteristics, tumor subtype, clinicopathological variables, treatment information, follow-up, and outcome.

**Sheet 2. DataDictionary**

Data dictionary describing all variables included in the *ClinicalData* sheet. Variable definitions, coding schemes, abbreviations, and categorical values are provided to facilitate interpretation of the clinical dataset.

**Sheet 3. QuPath-derived composition**

Quantitative tissue composition measurements obtained from QuPath digital pathology annotations for each melanoma specimen. Reported values correspond to the percentage of tumor area, extracellular matrix (ECM), and tumor-infiltrating lymphocytes (TILs) within the annotated tissue region.

**Sheet 4. Kruskal_Dunn_Results**

Results of post hoc Dunn's multiple comparison tests following Kruskal–Wallis analysis evaluating pairwise differences in tumor content, ECM, and TIL percentages among melanoma subtypes. Reported values include the standardized test statistic (Z), unadjusted P values (P.unadj), and Benjamini–Hochberg adjusted P values (P.adj).

**Sheet 5. PERMANOVA**

Results of permutational multivariate analysis of variance (PERMANOVA) performed using tumor content, ECM, and TIL percentages to evaluate overall differences in tissue composition among melanoma subtypes. Reported values include degrees of freedom (Df), sums of squares (SumOfSqs), proportion of explained variance (R²), pseudo-F statistic (F), and permutation-based P value [Pr(>F)].

**Supplementary Table S2. Proteomic datasets, DIA acquisition parameters, peptide-level evidence, and machine learning feature selection.**

**Sheet 1. Protein_matrix**

Protein group abundance matrix generated by DIA-NN from DIA-MS analysis. Rows correspond to protein groups and columns correspond to melanoma samples. Protein group intensities are reported as exported by DIA-NN prior to downstream data processing.

**Sheet 2. Processed_Matrix**

Processed protein abundance matrix used for downstream statistical analyses. Data were log₂-transformed, median normalized, and filtered to retain proteins quantified in at least 70% of samples.

**Sheet 3. Peptide_matrix**

Peptide-level quantification matrix exported from DIA-NN. Rows correspond to identified peptide precursors and columns correspond to melanoma samples. This dataset provides the peptide-level evidence supporting protein group quantification, including the non-isoform-specific peptides reported for ATP5IF1.

**Sheet 4. DIAcycles**

Variable isolation window scheme used for DIA data acquisition on the Q Exactive HF-X mass spectrometer. The table lists the m/z range of each DIA isolation window used throughout the acquisition cycle.

**Sheet 5. SelectedGenesML**

Proteins selected by supervised machine learning approaches (Random Forest, LASSO regression, and feseR) as subtype-discriminative features. The table reports the proteins identified by each method and the consensus feature set used for downstream classification and visualization analyses.
